# Predictors and Moderators of Post-Treatment Depression Severity After Hypnotherapy and Cognitive Behavioral Therapy in an RCT for Mild-to-Moderate Depression

**DOI:** 10.3390/bs16091584

**Published:** 2026-09-07

**Authors:** Anna-Lena Brockmann, Lucia Pfeiffer, Anil Batra, Kristina Fuhr

**Affiliations:** Department of Psychiatry and Psychotherapy, Tübingen Center for Mental Health (TüCMH), University of Tübingen, Calwer Str. 14, 72076 Tübingen, Germanyanil.batra@med.uni-tuebingen.de (A.B.)

**Keywords:** depression, psychotherapy, hypnotherapy, cognitive behavioral therapy, predictor, RCT

## Abstract

Depression is one of the most prevalent mental health conditions, and cognitive behavioral therapy (CBT) is one of the most efficacious treatments for depressive disorders. Several studies have demonstrated the efficacy of hypnotherapy (HT) in treating depression. However, it remains unclear which treatment works the best for each patient. This study investigated global and treatment-specific predictors of CBT and HT in a secondary analysis of an RCT for patients with mild-to-moderate major depression. A total of 152 patients were randomly assigned to receive either HT (n = 74) or CBT (n = 78) as an individual outpatient treatment for six months. Both treatments were comparably effective. Baseline characteristics including sociodemographic, depression-related, and psychological variables were assessed. The primary outcome was the clinician-rated Montgomery–Åsberg Depression Rating Scale (MADRS) scores after the treatment. Predictors were examined using correlation, regression, and moderation analyses for hypothesis-generating purposes. The results did not support the hypothesized cognitive and behavioral variables predicting post-treatment depression severity for CBT nor hypnotic susceptibility or expectations predicting outcome for HT. We found that lower baseline depression severity and higher self-efficacy predicted a better overall treatment outcome for all patients. As exploratory results, antidepressant medication and environmental quality of life predicted treatment outcome for patients receiving HT. In patients with CBT, educational level and psychological quality of life were found as exploratory predictors of treatment outcome. These results should be considered preliminary before strong conclusions can be drawn. Future research should further investigate HT and refine predictor models more strictly.

## 1. Introduction

In the year 2019, around 280 million people worldwide were diagnosed with a depressive disorder (WHO, 2021). Of the years lived with disability, depressive disorders were second among all diseases and highest for mental health conditions in 2021 (The Lancet Psychiatry, 2024; GBD 2021 Diseases and Injuries Collaborators, 2024). There are various treatments for depressive disorders, including antidepressant medication (ADM) and psychotherapy. Cognitive behavioral therapy (CBT) and interpersonal psychotherapy (IPT) are considered to be the most evidence-based forms of psychotherapy (e.g., Hautzinger, 2008; Luty et al., 2007). Essential elements of CBT are behavioral activation (e.g., Ekers et al., 2014), cognitive therapy and modifying automatic thoughts (Jacobson et al., 1996), in addition to elements like psychoeducation, social skill training, and relapse prevention (J. S. Beck, 2020). At least ten sessions are recommended for CBT for depression to increase its efficacy (Honyashiki et al., 2014). For those with relatively severe depression, especially, a treatment length of 16 sessions improved treatment outcomes compared to those with milder symptoms (Shapiro et al., 1994). When similar effective treatments are available, for example, IPT and CBT (Lemmens et al., 2015), the question arises as to which works better for whom (Cohen & DeRubeis, 2018). Consequently, predictors and moderators for differential treatment success should be identified (Simon & Perlis, 2010; Kazdin, 2007, 2009) to reduce individual suffering, time, and direct and indirect costs. Sociodemographic variables such as age (Fournier et al., 2009), marital status (Barber & Muenz, 1996), gender, and socioeconomic status (Fournier et al., 2009) were found to predict outcome. Regarding disorder-related characteristics such as the duration of the current episode (Blom et al., 2007) and the severity of the episode before the start of treatment (Hamilton & Dobson, 2002), there is evidence for a negative influence on the outcome. The quality of life or expectations regarding the efficacy of a treatment are also related to the outcome (Fournier et al., 2009, Beard et al., 2016); higher expectations were related to a better treatment outcome (Beard et al., 2016; Webb et al., 2013). A high self-efficacy and the belief of a person in their own capacities can have positive effects on the course of the depression (Schindler & Körkel, 1994; Brenninkmeijer et al., 2019). Taking ADM against depression when starting with psychotherapy may also influence outcomes. Several studies and meta-analyses found that a combined treatment of psychotherapy with ADM is superior to stand-alone treatments for moderate-to-severe depression (Cuijpers et al., 2014; Cuijpers et al., 2020; Nemeroff et al., 2003).

### 1.1. Treatment-Specific Outcome Predictors for CBT

Within the theoretical framework of CBT, cognitive and behavioral variables, for example, a lack of reinforcement (Lewinsohn, 1974), were hypothesized to moderate or mediate the outcome of a treatment for depression. As one of the cognitive variables, rumination, which refers to thinking about negative events and experiencing negative thoughts in loops, can predict higher symptoms after the end of a treatment (Jones et al., 2008; Schmaling et al., 2002; Teismann et al., 2008) and lowers the probability for a remission from depression (Jones et al., 2008). Similarly, dysfunctional attitudes assessed before treatment can predict a worse outcome after treatment (Hamilton & Dobson, 2002).

### 1.2. Hypnotherapy in the Treatment of Depression

While there is notable evidence for the successful treatment of depression with CBT, hypnotherapy (HT) for depression has not yet been significantly researched within the gold standard of randomized controlled trials (RCTs). Hypnotherapy (HT) covers a broad range of techniques such as directive suggestion, ego-strengthening, Ericksonian approaches, imagery-based work, age progression or regression, resource activation, cognitive hypnotherapy, and others. Previous RCTs compared HT as an add-on to other treatments, for example, CBT. Meta-analyses showed additive effects of HT compared to CBT alone and also for depressive symptoms (Kirsch et al., 1995; Ramondo et al., 2021). However, only a few RCTs were published with samples of patients with a clinically confirmed diagnosis of depression (Alladin & Alibhai, 2007; Butler et al., 2006, Chiu et al., 2018; Fuhr et al., 2021). Only some of the previous RCTs studied HT as a stand-alone treatment (Chiu et al., 2018; Fuhr et al., 2021). The generalizability of RCT results and the usage of those designs in studies with HT can be questioned, for example, because of the challenges involved in manualizing the treatment (McCann & Landes, 2010). Nevertheless, decisions in the health care system as well as the development of treatment guidelines within Europe rely on RCTs and meta-analyses, as stated, for example, by the European Medicines Agency. Treatments lacking empirical evidence within the gold standard approach therefore remain “unseen” and excluded from standardized treatment protocols and recommendations. Even though the number of RCTs published during recent years has been increasing (see, for example, Ramondo et al., 2024; Çınaroğlu et al., 2025), the interpretation of results is insufficient, mainly due to small sample sizes or methodological shortcomings.

### 1.3. Treatment-Specific Outcome Predictors for Hypnotherapy

Hammond (2005)’s model suggests that different aspects such as cognitive and motivational variables, as well as a dissociative capacity, suggestibility, absorption and aspects of the therapeutic relationship, can influence outcomes of HT. In the literature, hypnotic susceptibility or suggestibility was considered as a main predictor for treatment outcome. Alladin and Alibhai (2007) found that higher hypnotic suggestibility was associated with better treatment outcomes in both CBT and HT patients. However, one very recent study found no moderation effect of hypnotic susceptibility for depression when comparing hypnosis with muscle relaxation (Siewert et al., 2026). Additionally, as suggested by the social cognitive theory for hypnosis put forward by Lynn et al. (2008), efficacy might also be influenced by expectancies, attributions, and motivations. Outcome expectation predicted better treatment outcomes in the study by Ramondo et al. (2024). HT outcome was also influenced by treatment expectancies and treatment credibility in patients with pain (Milling et al., 2007).

### 1.4. Aims of the Study

The aim of this study was to identify treatment-specific predictors and moderators of post-treatment depression severity after CBT and HT in mild-to-moderate depression in a secondary analysis based on the RCT of Fuhr et al. (2021). Regarding CBT, potential treatment-specific predictors were variables such as behavioral activation level and rumination. Regarding HT, hypnotic susceptibility was selected as a potential predictor or moderator. Since the outcome of both CBT and HT may also be influenced by self-efficacy and treatment expectations, those variables were considered to be global predictors. Other global predictors were sociodemographic, depression-related, and psychological variables such as quality of life. Predictors were identified in three steps. First, based on correlation analyses, potential predictors were identified for the overall sample and for a treatment with CBT and HT separately. Second, linear regression analyses were conducted to select the most prominent predictors. Third, a moderator analysis was conducted to predict treatment-specific moderators.

## 2. Materials and Methods

This secondary exploratory analysis was based on the RCT, which found that HT was non-inferior to CBT regarding the percentage symptom improvement after six months in patients with a depressive episode (Fuhr et al., 2017, 2021). The RCT followed the CONSORT and GCP guidelines and was conducted in accordance with the Declaration of Helsinki. Ethical approval was obtained by the Ethics Committee of the University Hospital Tuebingen (061/2015BO2, on 11 March 2015). The RCT was pre-registered (NCT02375308, ClinicalTrials.gov).

### 2.1. Participants

Patients with a current mild-to-moderate depressive episode confirmed with a diagnostic interview based on the Diagnostic and Statistical Manual of Mental Disorders—Fifth Edition (DSM-5, APA, 2013) were included in the RCT. In cases of concomitant ADM, a stable course of medication for the last three months or longer was necessary for inclusion. Patients with a lifetime diagnosis of bipolar disorder or schizophrenia, a diagnosis of chronic major depression, actual high suicidality, or other severe primary psychological disorders (active alcohol or drug dependence, actual post-traumatic stress disorder, or anorexia nervosa), or patients receiving outpatient psychotherapy within the last twelve months were excluded from the trial.

Overall, 152 patients were randomized 1:1 with blocks of 40 to 16–20 individual sessions of either HT or CBT. The treatment allocation was concealed from patients and therapists until just before the first treatment session. Most of the patients were female (n = 100, 65.8%), and 114 (75.0%) had a high-school degree. Mean age was *M* = 39.5 (*SD* = 14.6). Fifty-six (36.8%) of the patients were using concomitant ADM (dummy-coded yes/no), with mostly SSRIs being prescribed (41.1%). For the sample characteristics per intervention condition, see Table 1. Regarding the CONSORT flow and the different types of ADM, see Fuhr et al. (2021).

### 2.2. Interventions

Patients received individual outpatient psychotherapy in 16–20 sessions during a six-month period with a therapist who was specifically licensed and trained in the respective intervention. The CBT manual was based on the German treatment manual for CBT (Hautzinger, 2013). CBT included modules on psychoeducation, behavioral activation, cognitive restructuring and meta-cognitive strategies but also interpersonal competence and training problem-solving skills using different tasks, as well as homework. In terms of relaxation techniques, only progressive muscle relaxation was allowed in CBT. The HT manual was based on a collection of different modules published in German (Wilhelm-Goessling et al., 2020). The HT treatment was based on the psychodynamic model of the ego-structure by Rudolf (2013). Hypnotic elements should be more directive with patients with a lower ego-structure, while patients with a higher ego-structure might benefit from open techniques and suggestions. HT included modules on resource activation, processing and integrating positive and negative experiences from the past, and the development of positive solution imagery. Hypnotic inductions were conducted individually by therapists with their patients. Patients were encouraged to practice self-hypnosis outside the sessions using self-hypnosis skills that were developed with the therapist. Alternatively, the hypnotic trances were recorded by each patient to practice at home. Details regarding the training of therapists and manual adherence in the RCT have been described previously (Fuhr et al., 2021). All therapists had at least three years of experience with the respective treatment method and were trained in one (CBT) or two (HT) days. Supervisions were held monthly with an expert in the field and a treatment manual. To allow for the tailoring of both treatments, all therapists were allowed to select the sequence of treatment modules according to the patient’s needs rather than following a rigid protocol. To assure manual adherence by the therapists, four external raters were involved in assessing the treatment fidelity. Results are published in Fuhr et al. (2021). There was a certain “overlap” in the CBT and HT treatment, for example, regarding the current symptoms and topics, individual backgrounds of patients, and aspects of motivational interviewing. CBT covered psychoeducation, behavioral activation, cognitive techniques, concrete plans, agenda setting, homework, and SORC analysis. HT techniques included resource activation, for example, via a safe place or imagining a resourceful animal, using direct or indirect trance inductions, association or dissociation techniques, seeding, time progression and regression, posthypnotic suggestions, externalizing symptoms, using ideomotor signals, and utilizing the specific symbols and wording of the patient. The treatment fidelity items are available in German upon request from the authors.

### 2.3. Outcome Measure

The Montgomery–Åsberg Depression Rating Scale (MADRS, Montgomery & Asberg, 1979) was used. Depressive symptoms were assessed with the clinician-rated MADRS at baseline and after treatment. The MADRS shows a high clinician interrater reliability (Montgomery & Asberg, 1979) as well as external validity with other depression and a high sensitivity to change (Carmody et al., 2006; Davidson et al., 1986). Raters were blinded regarding the intervention of the patient. The MADRS score after treatment was used as the outcome variable for all analyses.

### 2.4. Sociodemographic Variables

Variables such as gender, age, and educational level were assessed via self-report at baseline.

### 2.5. Depression-Related Variables

The duration of the current depressive episode, the lifetime history of previous episodes and the presence of comorbid mental health conditions (yes/no) was assessed at baseline in a clinician-based interview (First et al., 1997).

### 2.6. Psychological Variables

The global functional level was measured with the 12-item version of the WHO Disability Assessment Schedule (WHODAS, WHO, 2012), which has good external validity and internal consistency within Cronbach’s *α* = 0.83–0.92 (Federici et al., 2017; Axelsson et al., 2017):

Quality of life concerning physical health, psychological health, social relationships, and environment was assessed with 26 items with the WHO Quality of Life (WHOQOL-BREF, The WHOQOL Group, 1998). The WHOQOL has acceptable internal consistencies (Cronbach’s *α* = 0.82–0.95) and a good construct validity (Bonomi et al., 2000).

The behavioral activation levels of patients were measured with the Behavioral Activation for Depression Scale (BADS, Kanter et al., 2007) with 25 items, reporting the total scale but also the four subscales of activation, avoidance/rumination, work/school impairment, and social impairment. The BADS has good internal consistency and construct validity (Kanter et al., 2009).

Self-efficacy was assessed with the ten-item General Self-Efficacy Scale (GSES, Schwarzer & Jerusalem, 1995), which shows good external validity among different cultures (Luszczynska et al., 2005).

Hypnotic susceptibility was assessed with the Harvard Group Scale of Hypnotic Susceptibility (HGSHS, Shor & Orne, 1962) in an individual session for each patient with an audio-tape-recorded German version (Bongartz, 1985) followed by ten self-reported questions.

Despite random allocation to the treatment, we asked patients before randomization to rate their expectations regarding the efficacy of both treatments separately with a visual analogous scale (0–100).

Characteristics of the total sample and for each treatment condition separately are displayed in Table 1.

### 2.7. Statistical Analysis

Statistical analyses were performed with IBM SPSS Statistics Versions 27 and 28.

The analyses were conducted with all randomized patients (*N* = 152) based on an intention-to-treat (ITT) analysis with the post-treatment depression severity score.

Missing data in the primary outcome MADRS occurred in 18 patients, either due to a declination to participate after randomization (*n* = 1), a discontinuation of the study treatment before 16 sessions (*n* = 10), or not being available for the outcome assessment (*n* = 7). After ensuring that the missing data of the post-treatment MADRS scores were at random with the Little MCAR test, we replaced missing data with the multiple imputations method based on the linear regression imputation algorithm automatically generated by SPSS. The pooled descriptive data of the five imputations are reported and were used for the subsequent analyses. Per-protocol sensitivity analysis regarding the primary outcome can be found in Fuhr et al. (2021).

Missing data also occurred in some sociodemographic, depression-related, and psychological variables at baseline. Four patients had up to two missing items in the GSES and one patient had one missing item in the WHODAS in the paper–pencil questionnaires. Those were replaced by the median to calculate the sum scores. If more than a 20% rate of missing data occurred, the number of participants was reduced for subsequent analyses.

To select variables for the regression analyses, bivariate Pearson’s or point-biserial correlations were first calculated between sociodemographic, depression-related, and psychological variables with the outcome MADRS after treatment. Correlation analyses were conducted in the total sample, as well as separately for HT and CBT patients. After assuring normal distribution, linearity, homoscedasticity, and checking for multicollinearity (*r* < 0.7), hierarchical and stepwise regression models were tested. In a first block, the symptom severity at baseline (assessed with MADRS) was entered as a variable. In a second block, the variables identified as potential predictors based on the regression analyses (*p* > 0.05) were included in a stepwise manner. Regression analyses were also calculated with the MADRS symptoms at baseline as an additional variable (not included as an extra block) to compare the relevance of the effects of symptom severity at baseline on the outcome.

To select potential moderators, variables which showed a significant correlation with the outcome in either HT or CBT were included in a moderator analysis with the PROCESS macro for SPSS based on Hayes (2018)’s method. The factor intervention (dummy-coded as HT = 0, CBT = 1) served as the independent variable, and the selected variables were included as the moderator variables. Moderator analyses were run separately for each variable.

## 3. Results

### 3.1. Selection of Variables in the Correlation Analyses

As displayed in Table 2, in the total sample, the symptom severity at baseline, the duration of the current episode, global functioning, self-efficacy expectations, and quality of life regarding psychological and environmental domain significantly correlated with depressive symptoms after treatment. In patients receiving CBT, despite the symptoms at baseline, a lower school education, higher global functioning, better psychological quality of life, higher behavioral activation level, and lower social impairment, as well as higher self-efficacy expectations, correlated with lower symptoms in the outcome. In patients receiving HT, higher symptom levels at baseline and treatment with ADM were associated with higher symptoms after treatment.

### 3.2. Global Predictors for Treatment Outcome in the Total Sample

In the regression model for the total sample, the variable duration of the current episode, global functioning, self-efficacy expectations, and quality of life regarding psychological and environmental domain were entered in the second block after the MADRS at baseline (first block). Due to some missing data in questionnaires, the total sample for the regression analysis was *n* = 141. The MADRS at baseline (*β* = 0.28, *t*(137) = 3.71, *p* < 0.001) as well as self-efficacy (*β* = −0.34, *t*(137) = −4.29, *p* < 0.001) and the duration of the current episode (*β* = 0.17, *t*(137) = 2.28, *p* = 0.024) were identified as predictors for the outcome. The model explained 20% of the variance (Δ*R*^2^ = 0.205, *F*(3,137) = 12.98; *p* < 0.001); see Table 3.

In the regression analysis with MADRS at baseline as an additional variable (not entered in block 1), the same predictors could be identified (GSES t1: *β* = −0.32, *t*(137) = −4.29, *p* < 0.001, MADRS baseline: *β* = 0.28, *t*(137) = 3.71, *p* < 0.001, duration of current episode: *β* = 0.17, *t*(137) = 2.28, *p* = 0.024). Overall, 20% of the variance could be explained (Δ*R*^2^ = 0.205, *F*(3,137) = 12.997; *p* < 0.001). Depressive symptoms at baseline were therefore not the primary predictor for symptoms after treatment.

### 3.3. Treatment-Specific Predictors for Treatment Outcome

The variables self-efficacy, global functioning, school education, and environmental quality of life were entered in the regression analysis for CBT. Since there was a problem of multicollinearity resulting in a high correlation (*r* > 0.7) of the BADS total score with some of the subscales, only the BADS total score was entered in the regression analysis in block 2. The sample consisted of *n* = 75 patients. The MADRS at baseline (*β* = 0.31, *t*(73) = 2.92, *p* = 0.005) and self-efficacy (*β* = −0.27, *t*(73) = −2.52, *p* = 0.014) were identified as predictors for the outcome. The model explained 15.9% of the variance (Δ*R*^2^ = 0.159, *F*(2,73) = 8.07; *p* < 0.001); see Table 4.

In the second regression analysis with MADRS at baseline as an additional variable, other predictors were identified (WHOQOL-psychological: *β* = −0.33, *t*(73) = −3.08, *p* = 0.003; school education: *β* = 0.25, *t*(73) = 2.33, *p* < 0.023) but not the MADRS at baseline or the GSES. Here, 15.2% of the variance could be explained (Δ*R*^2^ = 0.152, *F*(2,73) = 7.74; *p* < 0.001). Given that the two predictors were identified in the stepwise regression model, the role of initial symptom severity has to be questioned for CBT patients in this study.

In the first regression model for HT patients, ADM, self-efficacy expectations, and quality of life regarding psychological and environmental domain were entered in the second block. Due to some missing data in questionnaires, the total sample for the regression analysis was *n* = 69. The MADRS at baseline (*β* = 0.26, *t*(64) = 2.50, *p* = 0.015), self-efficacy (*β* = −0.26, *t*(64) = −2.48, *p* = 0.016), quality of life regarding the environmental domain (*β* = −0.23, *t*(64) = −2.12, *p* = 0.038), and ADM (*β* = 0.38, *t*(64) = 3.70, *p* < 0.001) were identified as predictors for the outcome. The model explained 33.7% of the variance (Δ*R*^2^ = 0.337, *F*(4,64) = 9.65, *p* < 0.001); see Table 5.

In the second regression analysis with MADRS as an additional variable, the same predictors were identified (MADRS at baseline: *β* = 0.26, *t*(64) = 2.50, *p* < 0.015; GSES: *β* = −0.26, *t*(64) = −2.48, *p* = 0.016; WHOQOL-environmental: *β* = −0.23, *t*(64) = −2.21, *p* = 0.038; ADM: *β* = 0.38, *t*(64) = 3.70, *p* < 0.001). Overall, 33.7% of the variance could be explained (Δ*R*^2^ = 0.337, *F*(4,64) = 9.65; *p* < 0.001). Since the same variance could be explained with the same predictors as before, it can be assumed that the outcome was not primarily predicted by symptoms at baseline.

### 3.4. Moderators for Treatment Outcome in CBT and HT

For the moderator analysis, the variables ADM, BADS total score, and school education were selected. The overall model of the first moderator analysis was significant, *F*(3,148) = 3.36, *p* = 0.020, and explained a variance of 6.38% (*R*^2^ = 0.064). The results showed that ADM significantly moderated the effect between the treatment condition and the outcome (Δ*R*^2^ = 6.05%, *F*(1,148) = 9.57, *p* = 0.002, 95% *CI* [−14.918, −3.288]). Treatment with ADM was associated with lower outcome (or higher depressive symptoms after treatment) in HT compared to CBT. In the moderator analysis with the BADS total score, no effects were found (Δ*R*^2^ = 2.98%, *F*(3,144) = 1.721, *p* = 0.165, 95% *CI* [−0.2405, 0.0619]). Finally, the overall model with the variables school education and treatment condition was significant, *F*(3,148) = 3.68, *p* = 0.014, with a variance of 6.40% (*R*^2^ = 0.064) explained. The results showed a significant moderation effect of school education (Δ*R*^2^ = 6.39%, *F*(1,148) = 7.95, *p* = 0.006, 95% *CI* [1.3479, 7.6603]). Higher school education was associated with lower treatment outcomes for patients receiving CBT, while the opposite effect was observed for HT.

## 4. Discussion

In this secondary analysis, we wanted to identify overall and treatment-specific predictors and moderators for treatment outcome with HT and CBT. Overall, we found that in addition to the negative influence of depressive symptoms at baseline on treatment outcome, self-efficacy was associated with better outcomes independent of the type of treatment and also in each treatment-specific regression analysis. However, we did not find support for treatment-related predictors such as cognitive and behavioral variables for CBT and hypnotic susceptibility or expectations for HT.

The negative effect of depressive symptoms at baseline on treatment outcome is already known (Hamilton & Dobson, 2002; Catarino et al., 2018). In severe depression in particular, the severity of depressive symptoms at baseline seems to be a consistent predictor for negative treatment outcome (Lyons & Delgadillo, 2025). Nevertheless, the small variance explained by depressive symptoms at baseline in our study underlines the relevance of other predictors.

The results of this study indicate that self-efficacy can be seen as a global predictor for outcome of psychotherapy for depression. This would be in line with Maddux and Meier (1995). Self-efficacy was found to enhance the probability of a treatment response in CBT for depressed patients (Stiles-Shields et al., 2015). In line with Alladin (2009) and Yapko and Criswell (2023), self-efficacy was also predictive for patients undergoing HT. This result indicates that HT techniques such as ego-strengthening (for example, by creating positive future images and integrating negative and positive experiences of the past; see also Alladin, 2009) could improve the course of depression during treatment for patients with higher self-efficacy at baseline. CBT is also characterized by providing a treatment rationale for the patient, as well as a transparent session structure, goal-oriented agenda setting, clear tasks and homework (A. T. Beck et al., 1979; J. S. Beck, 2020), and the idea of making the patient an expert on the disorder through psychoeducation and self-management (King & Boswell, 2019; Kanfer & Hagerman, 1981). One explanation for self-efficacy as a predictor for post-treatment severity could be that CBT as well as HT in our study contributed to the improvement in patients with high self-efficacy.

There was no association of rumination or behavioral activation with post-treatment outcome, either in the whole sample or in CBT. Rumination was considered crucial regarding relapse rates and recovery from depression (e.g., Michalak et al., 2011). However, in contrast to previous studies (Michalak et al., 2011; Upton et al., 2025), CBT treatment did not involve specific rumination-targeted techniques such as mindfulness or others, which could explain the absence of an effect related to cognitive factors. Nevertheless, behavioral activation was a substantial part of CBT treatment in this study, which is in line with the evidence (e.g., Ekers et al., 2014). We measured behavioral activation only via self-report and did not assess or track the actual “real” engagement of patients in activities. Therefore, we might not have been able to assess real changes in behavior with BADS.

The absence of a relationship between hypnotic suggestibility and treatment outcome could be explained by the type of HT used in the RCT. In the treatment with HT, a broad range of HT techniques was allowed. Therefore, HT not only included hypnotic techniques such as trances and imaginative work but also indirect techniques, seeding or finding metaphors, and externalizing the symptoms. The model behind the treatment manual was used mainly for the selection of treatment modules rather than the therapeutic rationale and background. The effect of the influence of suggestibility on the outcome of HT might have been influenced by the type of treatment. In Alladin and Alibhai (2007), for example, hypnosis was added to CBT, and some of the differential effects in their RCT might be explained by the suggestibility. Since HT is not financially covered by the health care system in Germany and is therefore considered a more “alternative” treatment for patients, the expectation of efficacy may have positively influenced the effect of the treatment with HT (Butler et al., 2006; Doering et al., 2018), which would be also in line with the social cognitive theory for hypnosis put forward by Lynn et al. (2008). In contrast, efficacy expectations were overall significantly higher for treatment with CBT than with HT in our study but were not significantly associated with treatment outcome. Nevertheless, there was a small correlation with outcome (r = −0.22) in patients with CBT, but expectations were not selected for the treatment-specific predictors or moderators, potentially due to the small power. HT is a treatment which focuses on reinstalling positive resources via imagination, time progression and association techniques (Yapko, 2006; Lankton & Matthews, 2010). Furthermore, depression is interpreted as a strategy that was helpful during the past to cope with interpersonal problems or difficult life events but is now no longer functional or in the patient’s volitional control (Yapko, 2006; Alladin, 2010). When integrated with CBT, HT can shape social and problem-solving skills and can contribute to better self-regulation strategies (see, for example, Yapko & Criswell, 2023). Future studies should also investigate global predictors for treatment outcome when both CBT and HT are integrated.

The lack of the support for the theory-driven variables in this study might also indicate that there are common factors (such as self-efficacy, expectations, or the therapeutic alliance) rather than therapy-specific ingredients that contribute to the outcome of psychotherapy independently of the type of treatment (see also Wampold, 2015). Despite assessing treatment fidelity and relying on treatment manuals, it could be that the some of the contextual factors (such as the therapeutic alliance, empathy, and treatment expectations) as well as the severity of the depression at baseline contributed more on the post-treatment severity than the expected treatment-specific variables. Nevertheless, previous research also failed to find consistent treatment-specific predictors for outcome, which could also be due to the different nature of study designs, sample sizes, and the complexity of the predictors (Eilertsen & Eilertsen, 2023).

As exploratory results, concomitant antidepressant medication was associated with poorer post-treatment outcome in the HT group in this exploratory secondary moderator analysis. This result is in contrast to most studies and meta-analyses, which found that severely depressed patients would benefit from psychotherapy with additional ADM (Cuijpers et al., 2014; Cuijpers et al., 2020). Similar to our results, some studies found the same for CBT without additional ADM (Lopez-Gomez et al., 2019; Whiston et al., 2019). Our result regarding ADM use, though, should be interpreted with care. ADM use was reduced to a binary variable despite different types of ADM being taken. Differences in depressive symptoms, educational level, prior treatment history, treatment resistance, chronicity, relapse risk, biological severity, comorbidity, patient preference, treatment expectations, and clinician judgment in patients taking ADM compared to those without ADM use might explain this.

While we found that patients with a lower level of education (lower than high school) showed better outcomes when receiving CBT instead of HT, other studies found that a higher level predicted a better treatment outcome (Hirschfeld et al., 1998; van Beljouw et al., 2010) and is considered to be a protective factor regarding mental health conditions such as depression and anxiety (Bjelland et al., 2008). In our RCT, the sample showed an overall high level of education (75% had a high-school or university degree) and cannot be considered as a representative sample. The results should be interpreted with care.

### Limitations

Overall, it is important to note that this study is a secondary exploratory analysis of an RCT comparing HT with CBT in patients with mild-to-moderate depression. The results can only be interpreted with care and cannot be generalized to other populations beyond mild-to-moderate depression or to patients with severe or chronic depression and should be considered preliminary. With regard to sample size, our total sample with N = 141 fulfilled the rules of Green (1991), whereas the sample size should be N = 104 + m (number of predictors) for multiple linear regression. Regarding the subgroup analyses, the sample size was limited to 74 CBT and 68 HT patients. Another rule of thumb for regression analyses stated that the minimum size should be N = 20 + 5m. This could also be fulfilled by the subgroups because five variables were tested for their predictive value. Nevertheless, it is possible that smaller effects and correlations were not detected due to the lower power. Particularly with regard to the influence of expectation but also of behavioral activation on outcome, it can be assumed that the effect remained undetected due to the sample size. Furthermore, moderating effects were found for variables where the amount of information was artificially reduced to a binary variable and sample sizes in subgroups were small. Given that we select variables not only based on theoretical assumptions and treatment rationales but also based on correlation analyses in an explorative nature, there is also a risk of multiple testing, which can result in false-positive findings. We did not correct for multiple predictor and moderator analyses. Due to the stepwise regression, there could also have been an effect of overfitting. Finally, the influence of the predictors and moderators on treatment outcome is only of a correlative but not causal nature. To account for causal relationships or mediating effects, the predictive variables should have been additionally assessed during the treatment period. It is also important to note that it cannot be concluded that patients undergoing treatment with HT in a current depressive episode without ADM would show better effects when receiving HT instead of CBT. There could have been a confounding effect of ADM use, for example, with the severity of symptoms, the treatment history, and other clinical variables. The study was initially designed to compare HT with CBT in a non-inferiority RCT and not to measure explicit predictors and moderators. The study also lacks external validation.

## 5. Conclusions

This study provides first and preliminary results regarding predictors and moderators for treatment with HT compared with CBT. We did not find the hypothesized cognitive and behavioral variables predicting post-treatment depression severity for CBT nor hypnotic susceptibility affecting outcome for HT. Instead, we found self-efficacy and depressive symptoms at baseline to be relevant for both treatment outcomes. Since some of the findings were only found in subgroups with small sample sizes and the methods were used for hypothesis-generating purposes, future studies should follow a more rigid analysis plan, such as pre-defined predictor sets.

## Figures and Tables

**Table 1:** Sample characteristics, including sociodemographic, depression-related and psychological variables.

Variables		Total		CBT		HT		
Sociodemographic	*N*	*M*/*n* (*SD*/%)	*n*	*M*/*n* (*SD*/%)	*n*	*M*/*n* (*SD*/%)	*t*(1)/*χ*^2^(1)	*p*
Age	152	39.45 (14.55)	78	37.55 (14.50)	74	41.45 (14.43)	1.66	0.099
Gender, female	152	100 (68.5%)	78	51 (65.4%)	74	49 (66.2%)	0.01	0.914
High school education or higher yes/no	152	114 (75.0%)	78	52 (70.3%)	74	62 (79.5%)	1.72	0.190
**Depression-related**								
MADRS baseline	152	21.09 (5.48)	74	21.80 (5.33)	78	20.42 (5.57)	1.55	0.122
Duration current episode (in weeks)	148	26.19 (20.77, range 3–101)	71	28.44 (22.21)	77	24.12 (19.26)	1.27	0.207
Number previous episodes	138	2.89 (1.92, range 1–15)	74	2.71 (1.18)	69	3.07 (2.65)	−1.04	0.302
Comorbidity yes/no	152	68 (44.7%)	78	36 (48.6%)	74	32 (41.0%)	1.70	0.427
ADM yes/no	152	55 (36.2%)	78	25 (32.1%)	74	30 (40.5%)	1.19	0.276
**Psychological**								
WHODAS	148	16.04 (6.37)	76	15.68 (6.56)	72	16.42 (6.18)	0.70	0.486
WHOQOL-physical	149	54.40 (12.97)	77	52.52 (12.8)	72	56.42 (12.93)	1.85	0.066
WHOQOL-psychological	149	38.59 (12.48)	77	37.91(13.43)	72	39.32 (11.42)	0.69	0.490
WHOQOL-social	148	49.2 (18.68)	77	47.24 (17.63)	71	51.32 (19.65)	1.33	0.185
WHOQOL-environment	148	70.14 (14.08)	77	71.10 (13.60)	71	69.10 (14.6)	−0.86	0.389
BADS total	148	71.28 (17.61)	77	69.64 (18.61)	71	73.06 (16.42)	1.18	0.239
BADS Activation	148	16.47 (6.57)	77	16.18 (6.10)	71	16.79 (6.10)	0.56	0.576
BADS Rumination	148	23.95 (7.68)	77	24.99 (7.63)	71	22.83 (7.63)	−1.72	0.088
BADS Work Impairment	148	16.01 (6.10)	77	16.56 (6.55)	71	15.42 (5.55)	−1.13	0.259
BADS Social Impairment	148	12.86 (6.28)	77	12.69 (6.33)	71	13.06 (6.16)	0.36	0.721
GSES	146	22.79 (5.04)	76	22.62 (5.04)	70	22.99 (5.06)	0.44	0.661
HGSHS	144	5.97 (2.39)	75	6.19 (2.31)	69	5.74 (2.47)	−1.13	0.262
Expectation (0–100)	139	67.44 (16.84)	74	72.47 (17.83)	65	61.71 (17.26)	−3.96	<0.001

Notes: MADRS = Montgomery Åsperg Depression Rating Scale; WHODAS = World Health Organization Disability Assessment Schedule; WHOQOL-BREF = World Health Organization Quality of Life Instruments-BREF; BADS = Behavioral Activation for Depression Scale; GSES = General Self-Efficacy Scale; HGSHS = Harvard Group Scale of Hypnotic Susceptibility.

**Table 2:** Bivariate correlations between sociodemographic, depression-related and psychological variables with the MADRS score after treatment.

Variables		Total			CBT			HT	
Sociodemographic	*n*	*r*	*p*	*n*	*r*	*p*	*n*	*r*	*p*
Age	152	−0.104	0.204	78	−0.150	0.190	74	−0.062	0.602
Gender, female	152	−0.096	0.241	78	−0.062	0.587	74	−0.128	0.279
High school education or higher yes/no	152	0.005	0.948	78	0.295 *	0.019	74	−0.220	0.060
**Depression-related**									
MADRS baseline	152	0.291 **	<0.001	78	0.331 **	0.003	74	0.259 *	0.026
Duration current episode (in weeks)	148	0.163 *	0.045	77	0.108	0.332	71	0.210	0.078
Number previous episodes	138	−0.024	0.783	69	−0.043	0.726	69	−0.011	0.927
Comorbidity yes/no	152	0.022	0.354	78	−0.108	0.696	74	0.151	0.373
ADM yes/no	152	0.057	0.486	78	−0.201	0.078	74	0.289 *	0.012
**Psychological**									
WHODAS	148	0.188 *	0.022	76	0.231 *	0.044	72	0.147	0.215
WHOQOL-physical	149	−0.97	0.237	77	−0.142	0.217	72	−0.057	0.632
WHOQOL-psychological	149	−0.320 **	0.000	77	−0.344 **	0.002	72	−0.301 *	0.010
WHOQOL-social	148	−0.063	0.448	77	−0.046	0.693	71	−0.078	0.519
WHOQOL-environment	148	−0.268 **	0.001	77	−0.178	0.122	71	−0.350 **	0.003
BADS total	148	−0.145	0.078	77	−0.260 *	0.023	71	−0.022	0.857
BADS Activation	148	−0.153	0.064	77	−0.121	0.296	71	−0.190	0.112
BADS Rumination	148	0.128	0.122	77	0.222	0.052	71	0.036	0.763
BADS Work Impairment	148	0.051	0.537	77	0.130	0.259	71	−0.039	0.745
BADS Social Impairment	148	0.031	0.706	77	0.229 *	0.045	71	−0.165	0.170
GSES	146	−0.348 **	<0.001	76	−0.289 *	0.010	70	−0.405 **	0.001
HGSHS	144	−0.018	0.832	75	−0.022	0.851	69	−0.026	0.834
Expectation (0–100)	139	−0.158	0.063	74	−0.224	0.055	65	−0.122	0.333

Notes: MADRS = Montgomery Åsperg Depression Rating Scale; WHODAS = World Health Organization Disability Assessment Schedule; WHOQOL-BREF = World Health Organization Quality of Life Instruments-BREF; BADS = Behavioral Activation for Depression Scale; GSES = General Self-Efficacy Scale; HGSHS = Harvard Group Scale of Hypnotic Susceptibility. * *p* < 0.05, ** *p* < 0.01.

**Table 3:** Predictors for depressive symptoms after treatment in the total sample (*n* = 141).

				95% Confidence Interval	
Variables	Regression Coefficient B	Standard Error	Beta	Upper Limit	Lower Limit
(Constant)	14.61	4.40		5.913	23.299
MADRS baseline	0.45	0.12	0.28 ***	0.212	0.696
GSES	−0.60	0.14	−0.34 ***	−0.842	−0.311
Duration current episode	0.08	0.03	0.17 *	0.010	0.142

Notes: *R*^2^ = 0.222, Δ*R*^2^ = 0.205, *F*(3) = 12.10 ***; * *p* < 0.05, *** *p* < 0.001. MADRS = Montgomery Åsperg Depression Rating Scale, GSES = General Self-Efficacy Scale.

**Table 4:** Predictors for depressive symptoms after treatment in CBT (*n* = 75).

				95% Confidence Interval	
Variables	Regression Coefficient B	Standard Error	Beta	Upper Limit	Lower Limit
(Constant)	13.53			2.596	9.640
MADRS baseline	0.47	0.16	0.31 **	0.149	0.788
GSES	−0.45	0.18	−0.27 *	−0.809	−0.094

Notes: *R*^2^ = 0.181, Δ*R*^2^ = 0.159, *F*(2) = 8.07 ***; * *p* < 0.05, ** *p* < 0.01, *** *p* < 0.001. MADRS = Montgomery Åsperg Depression Rating Scale; GSES = General Self-Efficacy Scale.

**Table 5:** Predictors for depressive symptoms after treatment in HT (n = 69).

				95% Confidence Interval	
Variables	Regression Coefficient B	Standard Error	Beta	Upper Limit	Lower Limit
(Constant)	25.12			8.262	41.970
MADRS baseline	0.45	0.18	0.26 *	0.091	0.811
ADM	7.29	1.97	0.38 ***	3.358	11.230
GSES	−0.50	0.20	−0.26 *	−0.895	−0.096
WHOQOL-environmental	−0.93	0.44	−0.23 *	−1.806	−0.054

Notes: *R*^2^ = 0.376, Δ*R*^2^ = 0.337, *F*(4) =9.65 ***; * *p* < 0.05, *** *p* < 0.001. MADRS = Montgomery Åsperg Depression Rating Scale; ADM = antidepressant medication (yes/no); GSES = General Self-Efficacy Scale; WHOQOL = World Health Organization Quality of Life.

## Data Availability

All materials, data and analyses of the study are available on request from the corresponding author. The data are not publicly available due to privacy or ethical restrictions.

## Abbreviations

The following abbreviations are used in this manuscript:

ADM	Antidepressant medication
BADS	Behavioral Activation for Depression Scale
CBT	Cognitive–behavioral treatment
GSES	General Self-Efficacy Scale
HGSHS	Harvard Group Scale of Hypnotic Susceptibility
HT	Hypnotherapy
IPT	Interpersonal psychotherapy
MADRS	Montgomery–Åsberg Depression Rating Scale
RCT	Randomized controlled trial
WHO	World Health Organization
WHODAS	World Health Organization Disability Assessment Schedule
WHOQOL-BREF	World Health Organization Quality of Life Instruments-BREF
